# The Clock Drawing Test as a predictor of cognitive decline in non-demented stroke patients

**DOI:** 10.1007/s00415-021-10637-z

**Published:** 2021-06-06

**Authors:** Ilaria Cova, Francesco Mele, Federica Zerini, Laura Maggiore, Silvia Rosa, Valentina Cucumo, Michela Brambilla, Alessia Nicotra, Giorgia Maestri, Pierluigi Bertora, Simone Pomati, Leonardo Pantoni

**Affiliations:** 1 Neurology Unit, Luigi Sacco University Hospital, Milan, Italy; 2 Stroke and Dementia Lab, “Luigi Sacco” Department of Biomedical and Clinical Sciences, University of Milan, Via Giovanni Battista Grassi 74, 20157 Milan, Italy

**Keywords:** Stroke, Post-stroke cognitive impairment, Post-stroke dementia, Clock Drawing Test, Predictivity

## Abstract

**Background:**

The early detection of patients at risk of post-stroke cognitive impairment (PSCI) may help planning subacute and long-term care. We aimed to determine the predictivity of two screening cognitive tests on the occurrence of mild cognitive impairment or dementia in acute stroke patients.

**Methods:**

A cognitive assessment within a few days of ischemic or hemorrhagic stroke was performed in patients consecutively admitted to a stroke unit over 14 months by means of the Clock Drawing Test (CDT) and the Montreal Cognitive Assessment-Basic (MoCA-B).

**Results:**

Out of 191 stroke survivors who were non-demented at baseline, 168 attended at least one follow-up visit. At follow-up (mean duration ± SD 12.8 ± 8.7 months), 28 (18.9%) incident cases of MCI and 27 (18%) cases of dementia were recorded. In comparison with patients who remained cognitively stable at follow-up, these patients were older, less educated, had more comorbidities, a higher score on the National Institutes of Health Stroke Scale (NIHSS) at admission, more severe cerebral atrophy, and lower MoCA-B and CDT scores at baseline. In multi-adjusted (for age, education, comorbidities score, NIHSS at admission and atrophy score) model, a pathological score on baseline CDT (< 6.55) was associated with a higher risk of PSCI at follow-up (HR 2.022; 95% CI 1.025–3.989, *p* < 0.05) with respect to non-pathological scores. A pathological baseline score on MoCA-B (< 24) did not predict increased risk of cognitive decline at follow-up nor increased predictivity of stand-alone CDT.

**Conclusion:**

A bedside cognitive screening with the CDT helps identifying patients at higher risk of PSCI.

## Introduction

Stroke is the second leading cause of death and the third leading cause of disability worldwide [1]. The major burden of stroke concerns the chronic phase. In collective perception as well as in clinical trials, disability after stroke is mainly intended as physical disability, although a broader vision that includes evaluation of cognitive impairment is recommended [1] because more than half of stroke survivors experience post-stroke cognitive impairment (PSCI). In two-thirds of cases, PSCI corresponds to a mild cognitive impairment, in one third to dementia [2] and significantly impacts the quality of life of stroke survivors [3, 4]. PSCI is a clinical entity that encompasses all types of cognitive decline following an index stroke; it may occur immediately or in the first months after stroke. In some cases, PSCI severity can worsen over time [5], perhaps because of an underlying degenerative or micro-vascular pathology.

Early identification of patients at risk for PSCI is likely important for the assessment of subacute and long-term management strategies for stroke patients (e.g., rehabilitation, adherence to therapies and to risk factors control) [6, 7]. Handy cognitive instruments to systematically assess stroke patients at bedside would be useful to formulate a cognitive prognosis, but there is no consensus about the best tools to use [8].

Champod et al. suggested the use of clock drawing test (CDT) in the acute phase due to its predictivity of functional and cognitive outcomes one year after stroke [9]; the authors further advised to administer CDT in association with another screening test to compare predictive abilities. For this reason, we wanted to extend the results of the study of Champod by comparing the performances of CDT with those of Montreal Cognitive Assessment Basic (MoCA-B) [10]. The two test were administered during the acute phase of stroke with the aim of assessing their predictivity on the occurrence of cognitive decline at follow-up in previously non-demented adults. In comparison with the original MOCA test [11], MOCA-B is a more culturally neutral test and does not include the CDT as the original version; its subtests might be less influenced by language and grapho-motor impact.

## Methods

### Study population

Consecutive patients with acute ischemic or hemorrhagic stroke admitted to the stroke unit of the Luigi Sacco University Hospital were recruited between January 1, 2018, and February 28, 2019. All patients underwent an assessment of demographic and clinical parameters, including the Cumulative Illness Rating Scale (CIRS) [12] for the evaluation of pre-existing comorbidities, the National Institutes of Health Stroke Scale (NIHSS) [13] for the evaluation of stroke severity, and the modified Rankin Scale (mRS]) [14] at the time of admission and discharge from the stroke unit to evaluate global functioning before and after the acute event. A neuroimaging evaluation by means of CT scan or MRI allowed the definition of recent ischemic or hemorrhagic lesions, the quantification of leukoaraiosis (using the Wahlund scale with a score ranging from 0 to 30) [15] and of global atrophy (using the Pasquier scale with a score ranging from 0 to 39) [16]. We performed an evaluation of pre-stroke functional abilities and cognitive status through an interview with carers of patients and the compilation of the activities of daily living (ADL) [17], instrumental activities of daily living (IADL) [18] and the clinical dementia rating (CDR) [19] scales; for this latter, a global score of 0 was considered indicative of the absence of a previous cognitive impairment, a global score of 0.5 suggestive of a previous mild cognitive impairment (MCI) and a global score of ≥ 1 suggestive of a previous dementia [20]. The two selected cognitive screening tests (MoCA-B and CDT) were administered at bedside during the hospitalization. MoCA-B assesses the same cognitive domains as the original MoCA (executive functions, language, orientation, calculations, conceptual thinking, memory, visuoperception, attention and concentration) but detects cognitive impairment also in illiterate and lower educated subjects. MoCA-B takes 10–15 min to be administered as the original MoCA; MoCA-B score ranges from 0 to 30 and cut-off score to detect MCI is < 24/30 [10]. To perform CDT patients were asked to draw numbers, hands, and center of a standard clock within a drawn circumference of 11.7 cm, and to set the time at 06:05. CDT score was computed and adjusted for age as proposed by Caffarra et al. ranging from 0 to 13: a cut-off score < 6.55/13 indicates a pathological score [21].

A combined score (CS) based on the performances on both tests (MoCA-B and CDT) was derived as follows: a CS = 2 corresponds to a non-pathological (i.e., normal or borderline) performance on both tests (MoCA-B ≥ 24 and CDT ≥ 6.55), a CS = 1 indicates a pathological performance in one of the two tests (MoCA-B < 24 or CDT < 6.55), and a CS = 0 was attributed when a pathological performance was obtained on both tests (MoCA-B < 24 and CDT < 6.55).

Patients were re-evaluated by a neurologist blinded to baseline test scores at the follow-up visit which included a standard neurological examination, the assessment of functional status and abilities by means of the mRS, ADL and IADL, and the evaluation of cognitive status by means of CDR and the repetition of MoCA-B and CDT. The first follow-up visit was planned 3–4 months after acute stroke; subsequent follow-up visits were scheduled at different times according to individual clinical requirements. The presence of PSCI was evaluated at each follow-up visit, based on the evaluating neurologist’s best clinical judgment, and was categorized as MCI or dementia. MCI was defined as follows: (a) subjective cognitive complaint as reported by patients or affirmed by an informant; (b) objective cognitive impairment at neurological examination of higher cognitive functions and/or at cognitive tests; (c) essentially preserved basic activities of daily living and minimal impairment in complex instrumental functions; and (d) essentially preserved general cognitive functioning, defined as a CDR score = 0.5. Dementia required evidence of (a) objective cognitive impairment at neurological examination of higher cognitive functions and/or at cognitive tests; (b) impaired basic and instrumental activities of daily living due to the cognitive impairment; and (c) decline of general cognitive functioning (CDR scale score > 0.5). In case of discrepancy among cognitive complaint (for MCI patients), objective cognitive impairment and functional abilities, patients underwent an extensive neuropsychological examination.

The calculation of the follow-up time for each patient was equal to the interval between acute stroke and the last neurological follow-up, except in the case of occurrence of dementia in which case the time of diagnosis was considered.

Verbal informed consent was obtained from all patients or from legally authorized representatives before the study.

### Statistical analysis

Descriptive statistics were used to characterize demographic, clinical, neuropsychological, and neuroimaging features of the cohort. A univariate analysis (using Mann–Whitney *U* test for continuous variables and Chi-square test for categorical variables) was performed to assess the differences between the group of patients who attended the follow-up visit and those who were lost at follow-up. Among patients who completed the follow-up visit, we performed a subgroup analysis on those who did not have pre-stroke dementia. In this group of patients, we performed a univariate analysis (Mann–Whitney *U* test for continuous variables and Chi-square test for categorical variables) to test the differences in baseline clinical, neuropsychological, and neuroimaging variables between those who did and those who did not show a decline of cognitive status at follow-up. Decline of cognitive status was defined as the transition from the absence of pre-stroke cognitive impairment to any degree of PSCI (MCI or dementia), or the transition from pre-stroke MCI to post-stroke dementia. Person years of follow-up were calculated by multiplying the number of non-demented patients who attended the follow-up by the duration (years) of each patient follow-up. The relative risk of decline of cognitive status in relation to score at CDT and MoCA-B (each stand-alone or in combination) was assessed by means of Cox proportional-hazard regression models to estimate hazard ratios (HRs) with 95% confidence intervals (CIs). Unadjusted and adjusted models (for variables statistically significant at the univariate analysis) were carried out. All analyses were performed using SPSS 26 with *α* level of *p* < 0.05.

## Results

Two hundred fifty one patients were recruited during the study period. For 28 patients (11.2%), no information about pre-stroke cognitive impairment was available because of the absence of a caregiver during the stroke unit stay. Based on the CDR scale, 132 patients (52.6%) had no pre-stroke cognitive impairment, 59 patients (23.5%) had pre-stroke MCI, and 32 patients (12.7%) had pre-stroke dementia.

MoCA-B was administered in the acute phase to 189 patients (75.2%), CDT to 170 patients (76.7%). Main reasons for failure in complete administration of cognitive tests were in order of frequency: severe aphasia or dominant upper limb motor deficit, impaired consciousness, patient’s refusal, and presence of a language barrier. The mean interval (± SD) between stroke onset and cognitive screening was 4.4 days (± 2.8).

One hundred sixty eight patients (66.9%) attended at least one follow-up visit. Thirty patients (11.9%) died during hospital stay or before the first follow-up visit, and 19 patients (7.6%) were lost at follow-up due to admission to nursing homes after hospitalization. Table 1 shows baseline characteristics of the whole sample and of patients adherent and non-adherent to follow-up. Patients who attended follow-up were younger, more educated, less disabled (at baseline and at discharge), more frequently cognitively unimpaired and had less severe leukoaraiosis and atrophy scores. MoCA-B score was higher in patients attending follow-up visits, while CDT score was not significantly different between patients adherent and non-adherent to follow-up. We had information about pre-event cognitive status for 159 (94.6%) patients who attended the follow-up; 149 (93.7%) of them were not demented at baseline. During the 149 person-years of follow-up (minimum follow-up time 0.23, maximum 2.76, mean 1.07, median 0.92, IQR 0.33–1.67 years), there were 28 (18.9%) incident cases of MCI and 27 (18%) incident cases of dementia. Of these latter, 6 were normal at baseline and were diagnosed with MCI at an intermediate follow-up, 5 had already MCI at baseline, remained stable at an intermediate follow-up visit and became demented afterwards. Table 2 shows demographic, clinical and radiological characteristics of non-demented patients at baseline according to the cognitive outcome at follow-up. Patients who developed a cognitive decline at follow-up were older, less educated, had more comorbidities (assessed by CIRS), a higher score at NIHSS at admission, more severe cerebral atrophy (measured with the Pasquier scale), and lower MoCA-B and CDT scores at baseline.

**Table 1:** Baseline characteristics of the whole sample and of patients adherent and non-adherent to follow-up

	Total sample	Adherent to follow-up	Non-adherent to follow-up	*p* value
	(*n* = 251)	(*n* = 168)	(*n* = 83)	
Age (years)	76.2 ± 12.4	74.1 ± 11.9	80.3 ± 12.3	< 0.001 ^a^
Gender				
Female	129 (51.4)	81 (48.2)	48 (57.8)	0.097 ^b^
Male	122 (48.6)	87 (51.8)	35 (42.2)	
Education (years)	8.6 ± 4.2	8.9 ± 4.1	6.3 ± 5.1	0.009 ^a^
Prestroke mRS score				
≤ 2	212 (84.5)	156 (92.9)	56 (67.5)	< 0.001 ^b^
> 2	39 (15.5)	12 (7.1)	27 (32.5)	
CIRS total score	0.8 ± 0.4	0.7 ± 0.3	0.8 ± 0.4	0.050 ^a^
Type of event				
Ischemic stroke	230 (91.6)	158 (94.0)	72 (86.7)	0.049 ^b^
Hemorrhagic stroke	21 (8.4)	10 (6.0)	11 (13.3)	
NIHSS score at admission	7.1 ± 7.2	4.6 ± 4.7	12.4 ± 8.5	< 0.001 ^a^
Wahlund scale score	8.1 ± 5.4	7.4 ± 5.4	9.5 ± 5.2	0.002 ^a^
Pasquier scale score	15.1 ± 8.0	13.4 ± 7.3	18.5 ± 8.3	< 0.001 ^a^
Prestroke cognitive impairment based on CDR scale				
Not available data	28 (11.2)	9 (5.4)	19 (22.9)	< 0.001 ^b^
Cognitively unimpaired (CDR = 0)	132 (52.6)	102 (60.7)	30 (36.1)	
Mild cognitive impairment (CDR = 0.5)	59 (23.1)	47 (28.0)	12 (14.5)	
Dementia (CDR ≥ 1)	32 (12.7)	10 (5.9)	22 (26.5)	
Baseline MoCA-B score	20.5 ± 6.1	21.1 ± 5.6	17.4 ± 7.5	0.012 ^a^
Baseline CDT score	8.4 ± 3.6	8.7 ± 3.3	7.1 ± 4.4	0.109 ^a^
mRS score at discharge				
≤ 2	147 (58.6)	128 (76.2)	19 (22.9)	< 0.001 ^b^
> 2	104 (41.4)	40 (23.8)	64 (77.1)	

Data are expressed as mean ± SD or number of observations (% of total observation)

*CDR* Clinical Dementia Rating Scale, *CDT* clock-drawing test, *CIRS* Cumulative Illness Rating Scale, *MoCA-B* Montreal Cognitive Assessment-Basic, *mRS* modified Rankin scale, *NIHSS* National Institutes of Health Stroke Scale

^a^Mann–Whitney’s *U* test

^b^Pearson’s Chi-square test

**Table 2:** Characteristics of pre-stroke non-demented patients (*n* = 149) by cognitive decline at follow-up

	Cognitively stable at FU (*n* = 94)	Cognitively declined at FU (*n* = 55)	*p* value
Age (years)	70.5 ± 12.6	78.4 ± 8.5	< 0.001 ^a^
Gender			
Female	53	32	0.086 ^b^
Male	41	23	
Education (years)	10.2 ± 3.9	7.1 ± 3.3	< 0.001 ^a^
Prestroke mRS score			
≤ 2	92 (97.9)	52 (94.6)	0.277 ^b^
> 2	2 (2.1)	3 (5.4)	
CIRS total score	0.6 ± 0.3	0.8 ± 0.3	0.008 ^a^
Type of event			
Ischemic stroke	90 (95.7)	51 (92.7)	0.430 ^b^
Hemorrhagic stroke	4 (4.3)	4 (7.3)	
Location of lesions			
Dominant hemisphere	60 (63.8)	30 (54.5)	0.338 ^b^
Non-dominant hemisphere	27 (28.7)	21 (38.2)	
Bilateral	7 (7.4)	3 (5.5)	
Territory of ischemic stroke lesions			
Anterior circulation	58 (65.2)	34 (68.0)	0.742 ^b^
Posterior circulation	29 (32.6)	14 (28.0)	
Both anterior and posterior circulation	2 (2.2)	2 (2.2)	
NIHSS score at admission	3.7 ± 4.2	6.0 ± 5.2	0.001 ^a^
Wahlund scale score	6.4 ± 5.2	8.1 ± 5.3	0.057 ^a^
Pasquier scale score	11.7 ± 6.9	14.6 ± 7.0	0.011 ^a^
Baseline MoCA-B score	22.9 ± 4.8	18.3 ± 5.7	< 0.001 ^a^
n.a	2 (2)	4 (7)	< 0.001 ^b^
< 24	44 (47)	40 (73)	
≥ 24	48 (51)	11 (20)	
Baseline CDT score	9.9 ± 2.7	7.2 ± 3.2	< 0.001 ^a^
n.a	10 (10)	10 (18)	< 0.001 ^b^
< 6.55	13 (14)	25 (46)	
≥ 6.55	71 (76)	20 (36)	
CS			
≥ 1 n.a. score	10 (10)	10 (18)	< 0.001 ^b^
CS = 0 (MoCA-B < 24 and CDT < 6.55)	11 (12)	23 (42)	
CS = 1 (MoCA-B < 24 or CDT < 6.55)	39 (42)	16 (29)	
CS = 2 (MoCA-B ≥ 24 and CDT ≥ 6.55)	34 (36)	6 (11)	
mRS score at discharge			
≤ 2	79 (84.0)	39 (70.9)	0.057 ^b^
> 2	15 (16.0)	16 (29.1)	

Data are expressed as mean ± SD or number of observations (% of total observation)

*CDT* clock-drawing test, *CIRS* Cumulative Illness Rating Scale, *CS* Combined Score, *ES* Equivalent Score, *MoCA-B* Montreal Cognitive Assessment-Basic, *mRS* modified Rankin scale, *n.a.* not available, *NIHSS* National Institutes of Health Stroke Scale

^a^Mann–Whitney’s *U* test

^b^Pearson’s Chi-square test

A normal CDT score at baseline (≥ 6.55) has a negative predictive value of 0.7 in respect of a diagnosis of cognitive impairment (MCI or dementia), which means that 7 out of 10 patients who obtained a CDT normal score at baseline did not develop cognitive impairment at follow-up.

Unadjusted Cox analysis showed that patients with a pathological score on CDT (< 6.55) at baseline had a significantly increased risk of cognitive decline at follow-up in comparison with those with a CDT ≥ 6.55 (HR 3.100; 95%CI 1.704–5.637, *p* < 0.001) (Table 3). This finding was confirmed after adjusting for age, education, CIRS total score, NIHSS at admission, and Pasquier scale score (HR 2.022, 95% 1.025–3.989, *p* < 0.05). Unadjusted and adjusted Cox analysis did not show an increased risk of cognitive decline at follow-up in patients with a pathological score of MoCA-B (< 24) or a CS = 0 (MoCA-B < 24 and CDT < 6.55).

**Table 3:** Risk of cognitive decline according to cognitive screening at baseline

Cognitive screening at baseline	Cognitive decline at follow-up	
	Unadjusted model	Adjusted model
	(HR, 95% CI)	(HR, 95% CI)
CDT stand-alone		
CDT ≥ 6.55	1	1
CDT < 6.55	3.100 (1.704–5.637) ^#^	2.022 (1.025–3.989) *
MoCA-B stand-alone		
MoCA-B ≥ 24	1	1
MoCA-B < 24	1.937 (0.939–3.995)	0.687 (0.293–1.610)
CS		
CS = 2 (MoCA-B ≥ 24 and CDT ≥ 6.55)	1	1
CS = 1 (MoCA-B < 24 or CDT < 6.55)	4.188 (1.690–10.380) *	1.988 (0.646–6.119)
CS = 0 (MoCA-B < 24 and CDT < 6.55)	1.732 (0.676–4.437)	1.063 (0.364–3.103)

Model 1: unadjusted; Model 2: adjusted for age, education, CIRS total score, NIHSS at admission, Pasquier scale score

CDT clock-drawing test, MoCA-B Montreal Cognitive Assessment-Basic, CS Combined score

*Significant with *p* < 0.05

^#^Significant with *p* < 0.001

**Fig. 1:**
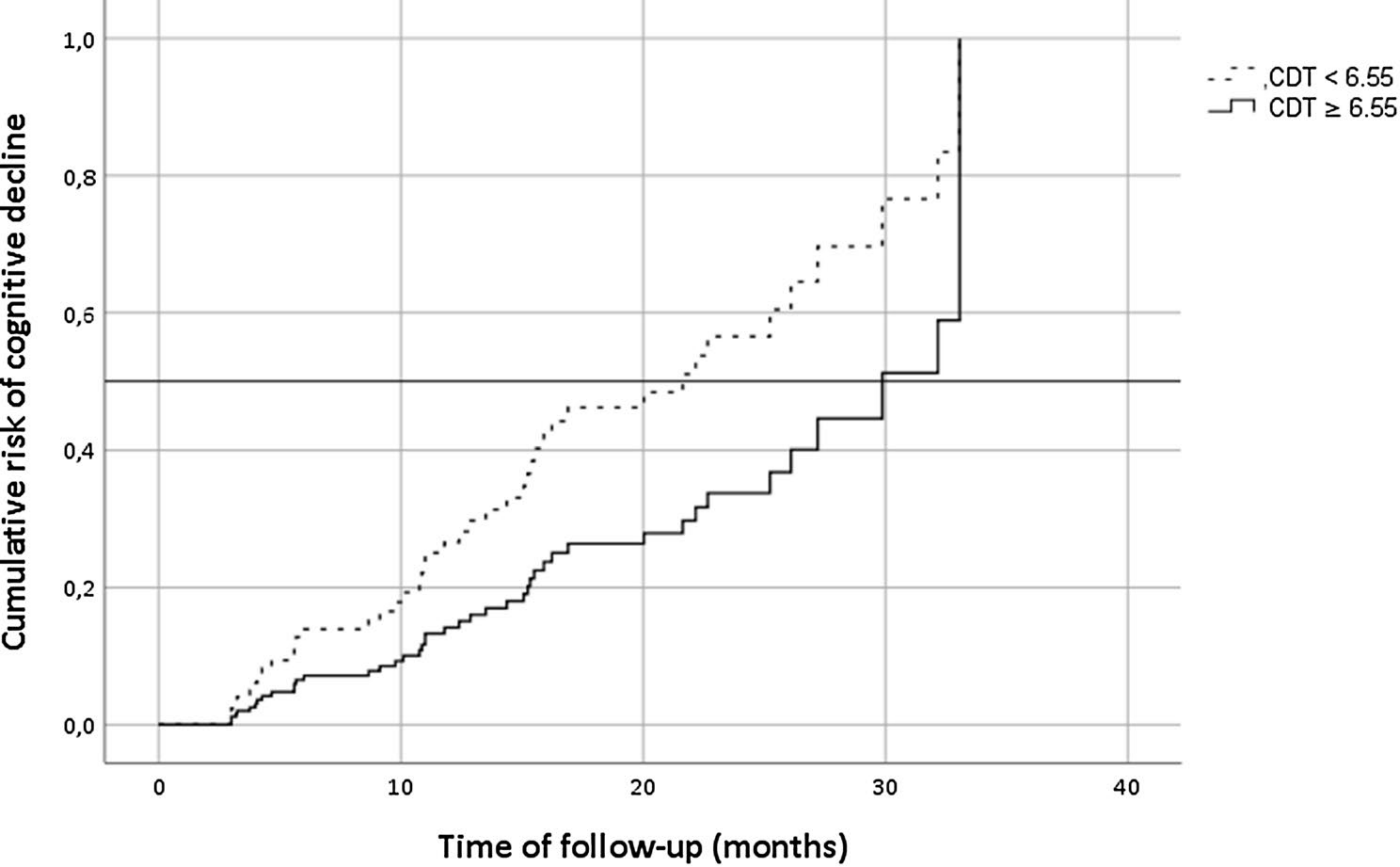
Cumulative risk curves of the effects of CDT baseline score (< 6.55 vs. ≥ 6.55) on cognitive decline. The figure is based on the adjusted model (for age, education, CIRS total score, NIHSS at admission, and Pasquier scale score) and reports data from patients reaching the end-point

Figure 1 shows the cumulative risk of cognitive decline according to the CDT baseline score (CDT < 6.55 vs. CDT ≥ 6.55).

The median time between the baseline assessment and the decline of cognitive status was 22 months in the group of patients with a pathological score at CDT (CDT < 6.55), 30 months for patients with a non-pathological score at on CDT (CDT ≥ 6.55); thus, a pathological score at CDT at baseline accelerates time of cognitive decline by about 8 months.

## Discussion

Findings from this study confirm that cognitive impairment affects a consistent number of patients suffering an acute cerebrovascular event. Bedside cognitive assessment in a patient with an acute neurological condition is cumbersome unless simple and reliable diagnostic tools are used. In this prospective hospital-based study, the presence of a pathological score on CDT in the acute phase of stroke doubles the risk of cognitive decline at follow-up in non-demented patients within 1 year.

Our findings are in agreement with those of Champod et al. [9] who found that the CDT administered in the acute phase of stroke was significantly associated with cognitive decline one-year post-stroke. Several different CDT scoring methods have been validated, but there is no consensus about which is the most accurate [22]. Two different scoring systems of CDT [11, 23] were used by Champod et al., but the more comprehensive system revealed a better predictive ability for a wider range of outcomes (ie, the Global Deterioration Scale, mRS, Barthel Index, and Reintegration to Normal Living index). Differently from the study of Champod et al., our study reports findings from clinical practice in which cognitive decline was defined based on best clinical judgment and follow-up were scheduled depending on the clinical needs of each patient.

The authors suggested comparing the predictive ability of the CDT to other screening measures in future studies. In our study, MoCA-B alone was not able to predict cognitive decline at follow-up and the combined use of CDT with MoCA-B did not provide a better predictability of cognitive decline in the follow-up, despite MoCA-B investigates other frequently impaired cognitive domains after stroke, such as attention, language, orientation, calculations, conceptual thinking, verbal memory, and concentration.

Several studies have investigated the utility of the MoCA [11] during the acute phase of stroke to predict cognitive outcome [24–28]. However, in the acute setting of stroke units, it is often difficult to assess patients due to a lack of time and resources; a very brief cognitive screening test such as CDT can be performed at bedside during a neurological examination in less than 5 min (unlike the MoCA which requires at least 10 min). Furthermore, CDT could be performed also with the non-dominant hand since its scoring is not influenced by an imperfect graphic, thus allowing patients with a plegic-dominant hand to complete the test.

The ability to identify patients at risk to develop cognitive dysfunction after stroke is important to set up cognitive rehabilitation in addition to physical rehabilitation, to predict future compliance to therapies, and to better advice caregivers about foreseen independence of patients after discharge.

Our study is not able to state whether the CDT is a screening tool that could be used also in other patient populations, for example those at high risk of dementia, or whether it is particularly suited for the stroke patient population. In this latter group, it has the advantage of the easiness of administration and, based on our results, of a good predictivity of cognitive outcome.

Our study has limitations. The diagnosis of pre-stroke cognitive impairment was based on the CDR score which was attributed on a history basis and mainly depended on the reliability of the caregiver's interview. Furthermore, in only a subgroup of patients, the diagnosis of cognitive decline after stroke was formulated after an exhaustive neuropsychological evaluation; in the other cases, the diagnosis was based on the neurologist’s best clinical judgment; it should be outlined, however, that resources for the thorough evaluation of cognitive functions of each stroke patients are not available in many stroke centers. A third limitation is the loss of patients seen at follow-up and the selection that therefore might have occurred. This could have limited the power to show associations between the baseline cognitive tests and cognitive decline in multivariate analyses, also because patients lost at follow-up were overall more diseased than those evaluated and, therefore, more likely to develop cognitive decline. Finally, it should be noted that normative data for MoCA-B on the Italian population are not available, and this may have led to underestimating the predictivity of this test.

Future studies could compare the predictive abilities of CDT to MoCA subtests to advise clinicians about the best 5-Minute Neuropsychological Protocol [29] to assess an early cognitive impairment in stroke units.

## Conclusion

Our study provides clinically relevant information for health care professionals working with stroke patients. Clinicians should be aware of the importance of a pathological performance at CDT in the acute phase of stroke as a predictor of cognitive decline at follow-up and consider the opportunity of administering a very brief cognitive screening at bedside of stroke patients to better plan rehabilitation program and expect longer-term post-stroke outcomes.

## Data Availability

Relevant data are within the paper. All the data that support the findings of this study are available on request from the corresponding author. The data are not publicly available due to privacy or ethical restrictions.

## References

[1] Katan M, Luft A (2018). Global burden of stroke. Semin Neurol.

[2] Barbay M, Diouf M, Roussel M (2018). Systematic review and meta-analysis of prevalence in post-stroke neurocognitive disorders in hospital-based studies. Dement Geriatr Cogn Disord.

[3] Park JH, Kim BJ, Bae H-J (2013). Impact of post-stroke cognitive impairment with no dementia on health-related quality of life. J Stroke.

[4] Sturm JW, Donnan GA, Dewey HM (2004). Quality of life after stroke: the North East Melbourne Stroke Incidence Study (NEMESIS). Stroke.

[5] Hu G-C, Chen Y-M (2017). Post-stroke dementia: epidemiology, mechanisms and management. Int J Gerontol.

[6] Eskes GA, Lanctôt KL, Herrmann N (2015). *Canadian *stroke best practice recommendations: mood, cognition and fatigue following stroke practice guidelines, update 2015. Int J Stroke.

[7] Quinn TJ, Elliott E, Langhorne P (2018). Cognitive and mood assessment tools for use in stroke. Stroke.

[8] Stolwyk RJ, O’Neill MH, McKay AJD, Wong DK (2014). Are cognitive screening tools sensitive and specific enough for use after stroke?: A systematic literature review. Stroke.

[9] Champod AS, Gubitz GJ, Phillips SJ (2019). Clock Drawing Test in acute stroke and its relationship with long-term functional and cognitive outcomes. Clin Neuropsychol.

[10] Julayanont P, Tangwongchai S, Hemrungrojn S (2015). The Montreal Cognitive Assessment-Basic: a screening tool for mild cognitive impairment in illiterate and low-educated elderly adults. J Am Geriatr Soc.

[11] Nasreddine ZS, Phillips NA, BÃ©dirian V,  (2005). The Montreal Cognitive Assessment, MoCA: a brief screening tool for mild cognitive impairment: MOCA: a brief screening tool for MCI. J Am Geriatr Soc.

[12] Salvi F, Miller MD, Grilli A (2008). A manual of guidelines to score the modified cumulative illness rating scale and its validation in acute hospitalized elderly patients. J Am Geriatr Soc.

[13] Brott T, Adams HP, Olinger CP (1989). Measurements of acute cerebral infarction: a clinical examination scale. Stroke.

[14] Farrell B, Godwin J, Richards S, Warlow C (1991). The United Kingdom transient ischaemic attack (UK-TIA) aspirin trial: final results. J Neurol Neurosurg Psychiatry.

[15] Wahlund LO, Barkhof F, Fazekas F (2001). A new rating scale for age-related white matter changes applicable to MRI and CT. Stroke.

[16] Pasquier F, Leys D, Weerts JGE (1996). Inter-and intraobserver reproducibility of cerebral atrophy assessment on MRI scans with hemispheric infarcts. Eur Neurol.

[17] Katz S, Ford AB, Moskowitz RW (1963). Studies of illness in the aged. The index of Adl: a standardized measure of biological and psychosocial function. JAMA.

[18] Lawton MP, Brody EM (1969). Assessment of older people: self-maintaining and instrumental activities of daily living. Gerontologist.

[19] Hughes CP, Berg L, Danziger WL (1982). A new clinical scale for the staging of dementia. Br J Psychiatry.

[20] Morris JC (1993). The clinical dementia rating (CDR): current version and scoring rules. Neurology.

[21] Caffarra P, Gardini S, Zonato F (2011). Italian norms for the Freedman version of the Clock Drawing Test. J Clin Exp Neuropsychol.

[22] Spenciere B, Alves H, Charchat-Fichman H (2017). Scoring systems for the Clock Drawing Test: a historical review. Dement Neuropsychol.

[23] Freedman M, Leach L, Kaplan E, Winocur G, Shulman K, Delis DC (1994). Clock Drawing: a neuropsychological analysis.

[24] Dong Y, Xu J, Chan BP-L (2016). The Montreal Cognitive Assessment is superior to National Institute of Neurological Disease and Stroke-Canadian Stroke Network 5-minute protocol in predicting vascular cognitive impairment at 1 year. BMC Neurol.

[25] Jacquin A, Binquet C, Rouaud O (2014). Post-stroke cognitive impairment: high prevalence and determining factors in a cohort of mild stroke. J Alzheimers Dis.

[26] Dong Y, Venketasubramanian N, Chan BP-L (2012). Brief screening tests during acute admission in patients with mild stroke are predictive of vascular cognitive impairment 3–6 months after stroke. J Neurol Neurosurg Psychiatry.

[27] Zietemann V, Georgakis MK, Dondaine T (2018). Early MoCA predicts long-term cognitive and functional outcome and mortality after stroke. Neurology.

[28] Salvadori E, Pasi M, Poggesi A (2013). Predictive value of MoCA in the acute phase of stroke on the diagnosis of mid-term cognitive impairment. J Neurol.

[29] Hachinski V, Iadecola C, Petersen RC (2006). National Institute of Neurological Disorders and Stroke-Canadian Stroke Network Vascular Cognitive Impairment Harmonization Standards. Stroke.

